# SIRT7 slows down stem cell aging by preserving heterochromatin: a perspective on the new discovery

**DOI:** 10.1007/s13238-020-00735-5

**Published:** 2020-05-20

**Authors:** Luyang Sun, Weiwei Dang

**Affiliations:** Huffington Center on Aging, Baylor College of Medicine, Houston, TX 77030 USA

Stem cell aging is one of the leading theories of aging and is gaining momentum in recent years with the development of new technologies to study stem cells (Schultz & Sinclair, 2016). In the stem cell aging theory, the age-associated physiological changes and functional declines at tissue and organismal levels are attributed to changes to various stem cell populations, both in their quantity and function. Hence, one of the pressing questions in the aging research is to understand the molecular mechanisms that preserve stem cell function and delay their aging process. In this issue, Bi, et al., reports a novel mechanism that slows down the aging process in human mesenchymal stem cells by preserving the structure of heterochromatin, which is mediated through the functions of SIRT7, one of the seven sirtuins found in all mammals (Bi et al., 2020).

Mammalian sirtuins are orthologs of yeast silent information regulator 2 (Sir2), an NAD^+^ dependent histone deacetylase that promotes genome stability and suppress the transcription at heterochromatin-like regions (Giblin et al., 2014). Since the first report on Sir2 that it extends budding yeast lifespan (Kaeberlein et al., 1999), great efforts have been made to explore the functions and mechanisms of sirtuins in aging and cellular senescence. The sirtuin family is evolutionarily conserved, from bacteria to mammals. Seven sirtuin members have been discovered in mammalian species, SIRT1-7. Although all of them contains a highly conserved NAD^+^- binding domain, their enzymatic activities, molecular functions, and cellular localizations vary greatly (Haigis & Sinclair, 2010).

SIRT7 is the only sirtuin that predominantly located in nucleolus. Early studies reveal that it is a regulator of rDNA transcription (Ford et al., 2006; Grob et al., 2009), and protein synthesis (Tsai et al., 2014). To date, numerous studies greatly enrich our understanding of SIRT7 functions. SIRT7 is a versatile regulator that involved in a wide range of biological processes including but not limited to genome instability, apoptosis, stress response, heterochromatin maintenance, DNA damage repair, mitochondrion homeostasis, cellular senescence and aging (Blank & Grummt, 2017; Wu et al., 2018). Majority of the SIRT7 functions are mediated via its enzymatic activities, such as deacetylation of histone H3K18 (Barber et al., 2012) and several nonhistone proteins, and desuccinylation of H3K122 (Li et al., 2016), although Paredes et al. report that SIRT7 extends HSC lifespan by stabilizing SNF2H at rDNA promoters without its catalytic activity (Paredes et al., 2018).

Consisted with its ortholog Sir2 in budding yeast, many lines of evidence showed that SIRT7 plays potent roles in cellular senescence and aging. SIRT7 deficient mice showed a shortened lifespan and aging related phenotypes (Vakhrusheva et al., 2008; Shin et al., 2013; Ryu et al., 2014; Mohrin et al., 2015; Vazquez et al., 2016). Loss of SIRT7 has been reported in a few of senescent cell lines (Mortuza et al., 2013; Lee et al., 2014; Ryu et al., 2014), and overexpression of SIRT7 in senescent-induced cells repress the expression of senescence markers such as p53 and p21 (Wronska et al., 2016).

However, in contrast to the versatility of SIRT7 functions, little was known about the molecular basis and pathways of SIRT7 during aging and cellular senescence, especially in human mesenchymal stem cells (MSCs), which attracted a lot of interests because of its potential in stem cell therapies and regenerative medicine (Freitag et al., 2016). In addition, it is known that heterochromatin loss and dysregulation occur during senescence (Chandra & Kirschner, 2016; Sun et al., 2018), but the exact mechanisms underlining MSC heterochromatin maintenance remain elusive.

Here, Bi et al. fills the gap in knowledge between loss of SIRT7 and heterochromatin dysregulation during cellular senescence in human MSCs. The authors report that SIRT7 antagonizes MSC senescence by silencing LINE1 retrotransposons at heterochromatin regions. Loss of SIRT7 leads to increased heterochromatin accessibility and up-regulation of LINE1 transcription, which in turn activates cGAS-STING pathway and autoimmune responses and eventually triggers MSC senescence (Bi et al., 2020).

The authors first show that loss of SIRT7 is a driver of senescence of human MSCs. In agreement with previous studies in other cell types, SIRT7 deficient MSCs have shorter replicative lifespan with accelerated senescence phenotypes, including slower growth rate, higher percentage of SA-β-gal-positive cells, higher ROS, and higher expression of senescence markers. Most importantly, these results demonstrate that SIRT7 is responsible for the maintenance of MSC heterochromatin at nuclear periphery by interacting with heterochromatin proteins and nuclear lamina.

By flag-tagged IP-mass spectrometry and co-IP assays, the authors identify a list of new SIRT7-interacting proteins including heterochromatin proteins KAP1, HP1α and HP1γ, and nuclear lamina proteins Lamin B1 and Emerin. Further experiments confirm that loss of SIRT7 resulted in lower protein levels of these proteins and increased accessibility of heterochromatin at the LINE1 promoter regions. These results, accompanied with the up-regulation of LINE1 transcription and increased retrotransposon activities, strongly indicate that SIRT7 represses LINE1 activation at human MSC heterochromatic regions. These findings shed new light on the function of SIRT7 and the molecular basis of MSC heterochromatin maintenance.

Finally, the authors complete the whole pathway by figuring out the downstream players of LINE1 in MSCs. Since LINE1 expression activates the cGAS-STING pathway and triggers cellular senescence in mice (de Cecco et al., 2019; Simon et al., 2019), the same pathway may be activated by LINE1 expression in human MSCs. Indeed, by multiple lines of evidence, including the gene expression profiles and the relevant protein abundant levels, derepression of LINE1 retrotransposons activates the DNA sensing cGAS-STING signaling pathway and auto immune responses in human stem cells.

In summary, the authors provide the first evidence that SIRT7 antagonizes cellular senescence in human MSCs through silencing LINE1 at heterochromatin regions. One of the critical results in the study is the discovery of heterochromatin proteins and nuclear lamina proteins as novel SIRT7-interacting proteins in human MSCs. Combined with the experiments showing the loss of SIRT7 causes increased heterochromatin accessibility and de-attachment of heterochromatin from nuclear periphery, this study functionally links SIRT7 to heterochromatin maintenance in MSCs.

Despite the recent progress, this study and other recent one prompt more questions regarding SIRT7 and its functions in heterochromatin and cellular aging. For example, how SIRT7 interacts with the heterochromatin structural proteins in human MSC remains to be investigated. Furthermore, other studies have showed that SIRT7 regulates heterochromatin via additional mechanisms. Li et al. reported that SIRT7 promotes chromatin condensation and DNA double-strand break repair by desuccinylation of H3K122 in various cancer cell lines (Li et al., 2016). Meanwhile, in human primary cells, SIRT7 maintains nucleolar heterochromatin by acting as a scaffold to stabilize SNF2H, a component of the heterochromatin silencing complex NoRC (Paredes et al., 2018). How these mechanisms influence one another remains to be studied.

Increased rDNA instability is a common senescence associated phenotype in human. As a mammalian homolog of Sir2 and the only sirtuin that primarily located at nucleoli where rDNA genes are enriched, it is possible that SIRT7 also antagonizes human MSC senescence via maintaining rDNA stability at heterochromatin regions. Indeed, Paredes et al. have reported that loss of SIRT7 resulted in derepression of heterochromatic rDNA gene clusters, with recombination and loss of rDNA gene copies, which in turn trigger acute cellular senescence (Paredes et al., 2018). In addition, SIRT7 is an epigenetic regulator that involved in various biological processes. Loss of SIRT7 in senescent cells may trigger multiple downstream pathways simultaneously. Besides of silencing LINE1 retrotransposons and rDNA gene clusters at heterochromatin regions, it has also been reported that SIRT7 represses mitochondrial unfolded protein responses and promote longevity of hematopoietic stem cells (Mohrin et al., 2015). Therefore, it will be interesting to see whether these downstream pathways of SIRT7 could be activated at the same time during senescence, and how these different pathways contribute to the cellular senescence in various tissues, as well as organismal aging in general.

## References

[CR1] Barber MF, Michishita-Kioi E, Xi Y, Tasselli L, Kioi M, Moqtaderi Z, Tennen RI, Paredes S, Young NL, Chen K (2012). SIRT7 links H3K18 deacetylation to maintenance of oncogenic transformation. Nature.

[CR2] Bi S, Liu Z, Wu Z, Wang Z, Liu X, Wang S, Ren J, Yap Y, Zhang W, Song M (2020). SIRT7 antagonizes human stem cell aging as a heterochromatin stabilizer. Protein Cell.

[CR3] Blank MF, Grummt I (2017). The seven faces of SIRT7. Transcription.

[CR4] Chandra T, Kirschner K (2016). Chromosome organisation during ageing and senescence. Curr Opin Cell Biol.

[CR5] de Cecco M, Ito T, Petrashen AP, Elias AE, Skvir NJ, Criscione SW, Caligiana A, Brocculi G, Adney EM, Boeke JD (2019). L1 drives IFN in senescent cells and promotes age-associated inflammation. Nature.

[CR6] Ford E, Voit R, Liszt G, Magin C, Grummt I, Guarente L (2006). Mammalian Sir2 homolog SIRT7 is an activator of RNA polymerase I transcription. Genes Dev.

[CR7] Freitag J, Bates D, Boyd R, Shah K, Barnard A, Huguenin L, Tenen A (2016). Mesenchymal stem cell therapy in the treatment of osteoarthritis: reparative pathways, safety and efficacy—a review. BMC Musculoskelet Disord.

[CR8] Giblin W, Skinner ME, Lombard DB (2014). Sirtuins: guardians of mammalian healthspan. Trends Genet.

[CR9] Grob A, Roussel P, Wright JE, McStay B, Hernandez-Verdun D, Sirri V (2009). Involvement of SIRT7 in resumption of rDNA transcription at the exit from mitosis. J Cell Sci.

[CR10] Haigis MC, Sinclair DA (2010). Mammalian sirtuins: biological insights and disease relevance. Annu Rev Pathol.

[CR11] Kaeberlein M, McVey M, Guarente L (1999). The SIR2/3/4 complex and SIR2 alone promote longevity in *Saccharomyces cerevisiae* by two different mechanisms. Genes Dev.

[CR12] Lee N, Kim DK, Kim ES, Park SJ, Kwon JH, Shin J, Park SM, Moon YH, Wang HJ, Gho YS (2014). Comparative interactomes of SIRT6 and SIRT7: implication of functional links to aging. Proteomics.

[CR13] Li L, Shi L, Yang S, Yan R, Zhang D, Yang J, He L, Li W, Yi X, Sun L (2016). SIRT7 is a histone desuccinylase that functionally links to chromatin compaction and genome stability. Nat Commun.

[CR14] Mohrin M, Shin J, Liu Y, Brown K, Luo H, Xi Y, Haynes CM, Chen D (2015). A mitochondrial UPR-mediated metabolic checkpoint regulates hematopoietic stem cell aging. Science.

[CR15] Mortuza R, Chen S, Feng B, Sen S, Chakrabarti S (2013). High glucose induced alteration of SIRTs in endothelial cells causes rapid aging in a p300 and FOXO regulated pathway. PLoS ONE.

[CR16] Paredes S, Angulo-Ibanez M, Tasselli L, Carlson SM, Zheng W, Li TM, Chua KF (2018). The epigenetic regulator SIRT7 guards against mammalian cellular senescence induced by ribosomal DNA instability. J Biol Chem.

[CR17] Ryu D, Jo YS, Lo Sasso G, Stein S, Zhang H, Perino A, Lee JU, Zeviani M, Romand R, Hottiger MO (2014). A SIRT7-dependent acetylation switch of GABPβ1 controls mitochondrial function. Cell Metab.

[CR18] Schultz MB, Sinclair DA (2016). When stem cells grow old: phenotypes and mechanisms of stem cell aging. Development.

[CR19] Shin J, He M, Liu Y, Paredes S, Villanova L, Brown K, Qiu X, Nabavi N, Mohrin M, Wojnoonski K (2013). SIRT7 represses myc activity to suppress er stress and prevent fatty liver disease. Cell Rep.

[CR20] Simon M, van Meter M, Ablaeva J, Ke Z, Gonzalez RS, Taguchi T, de Cecco M, Leonova KI, Kogan V, Helfand SL (2019). LINE1 derepression in aged wild-type and SIRT6-deficient mice drives inflammation. Cell Metab.

[CR21] Sun L, Yu R, Dang W (2018). Chromatin architectural changes during cellular senescence and aging. Genes.

[CR22] Tsai YC, Greco TM, Cristea IM (2014). Sirtuin 7 plays a role in ribosome biogenesis and protein synthesis. Mol Cell Proteomics.

[CR23] Vakhrusheva O, Smolka C, Gajawada P, Kostin S, Boettger T, Kubin T, Braun T, Bober E (2008). Sirt7 increases stress resistance of cardiomyocytes and prevents apoptosis and inflammatory cardiomyopathy in mice. Circ Res.

[CR24] Vazquez BN, Thackray JK, Simonet NG, Kane‐Goldsmith N, Martinez-Redondo P, Nguyen T, Bunting S, Vaquero A, Tischfield JA, Serrano L (2016). SIRT7 promotes genome integrity and modulates non‐homologous end joining DNA repair. EMBO J.

[CR25] Wronska A, Lawniczak A, Wierzbicki PM, Kmiec Z (2016). Age-related changes in sirtuin 7 expression in calorie-restricted and refed rats. Gerontology.

[CR26] Wu D, Li Y, Zhu KS, Wang H, Zhu W-G (2018). Advances in cellular characterization of the sirtuin isoform, SIRT7. Front Endocrinol.

